# Supplementary material for: Mapping responsive genomic elements to heat stress in a maize diversity panel
Source: Genome Biol. 2022 Nov 7;23:234. doi: 10.1186/s13059-022-02807-7 (PMC9639295; doi:10.1186/s13059-022-02807-7)
Supplement: Supplementary file 1 — Additional file 1: Supplementary figures of mapping responsive genomic elements to heat stress in a maize diversity panel. Fig S1-S9 include additional information of data quality evaluation, methods and associated results. [file 13059_2022_2807_MOESM1_ESM.docx]

**Additional file 1:** Supplementary figures (Fig S1-S9) of mapping responsive genomic elements to heat stress in a maize diversity panel.

**
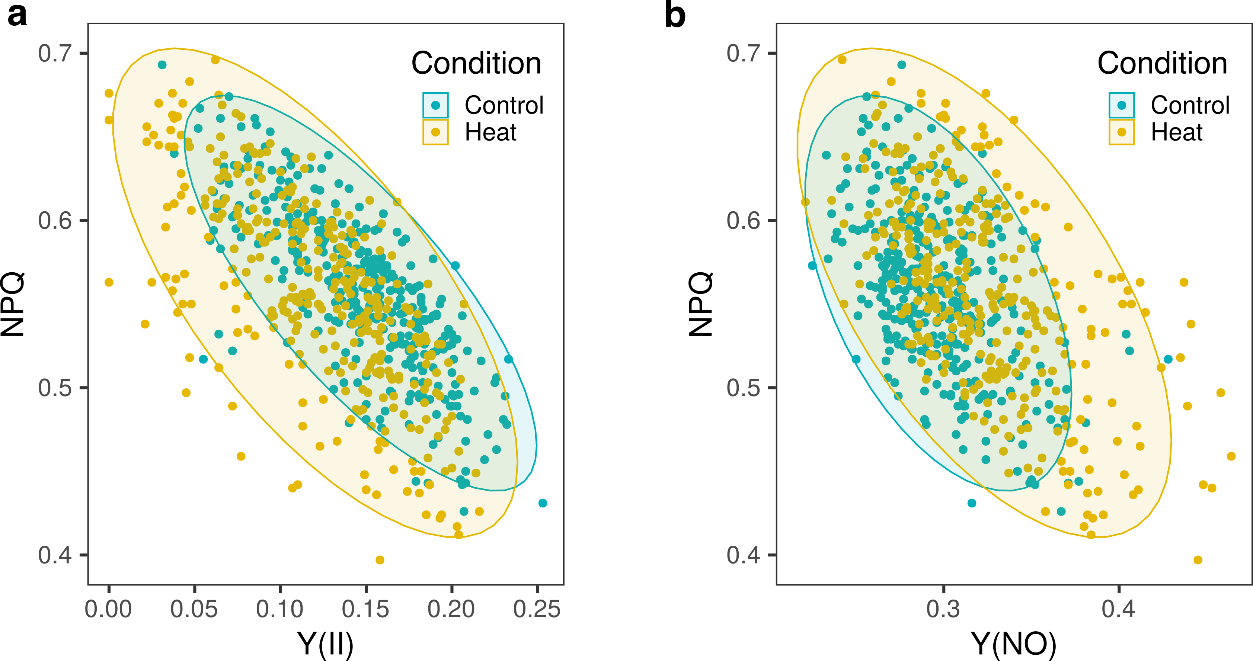
**

**Fig S1. Segregation between heat stressed plants and control plants using measured photosynthetic parameters.** (a) Heat stressed plants and control plants were segregated using Y(NPQ) and Y(II); (b) Heat stressed plants and control plants were segregated using Y(NPQ) and Y(NO).

**
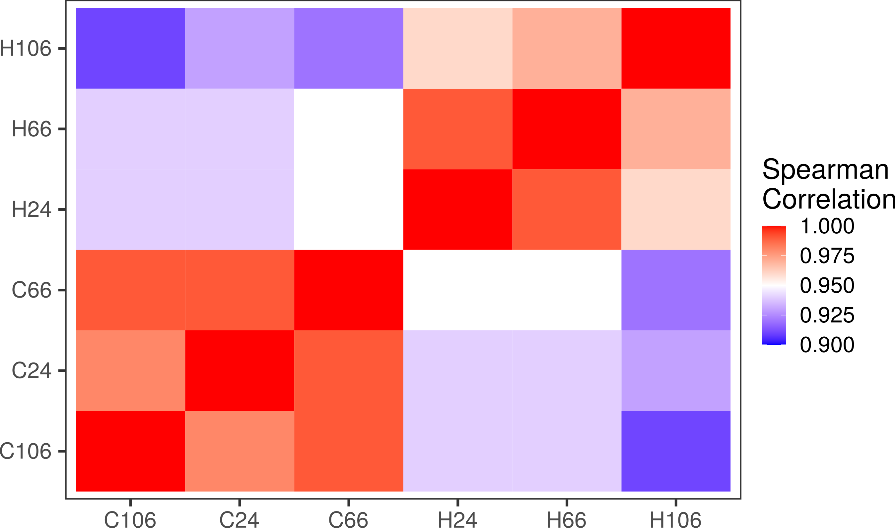
**

**Fig S2. Pairwise correlations between B73 samples employed in the large panel heat stress using normalized gene read counts.** Spearman correlation was employed for testing. Genotype code 24, 66 and 106 represented three biological replicates of B73 in both control (C) and heat (H) conditions.


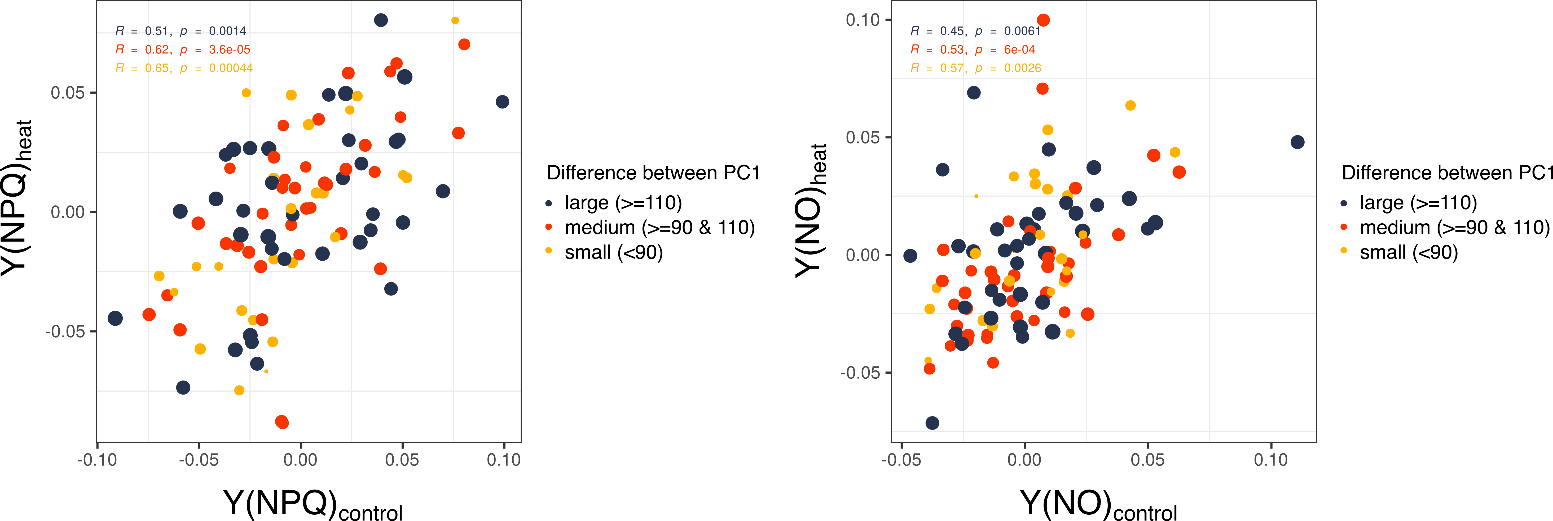


**Fig S3. Correlations between phenotypic and transcriptomic data.** Genotypes with variable levels of PC1 differences were split into three classes: large, medium and small. The correlation of BLUPs generated from Y(NPQ) and Y(NO) in control and heat condition was separately calculated.

**
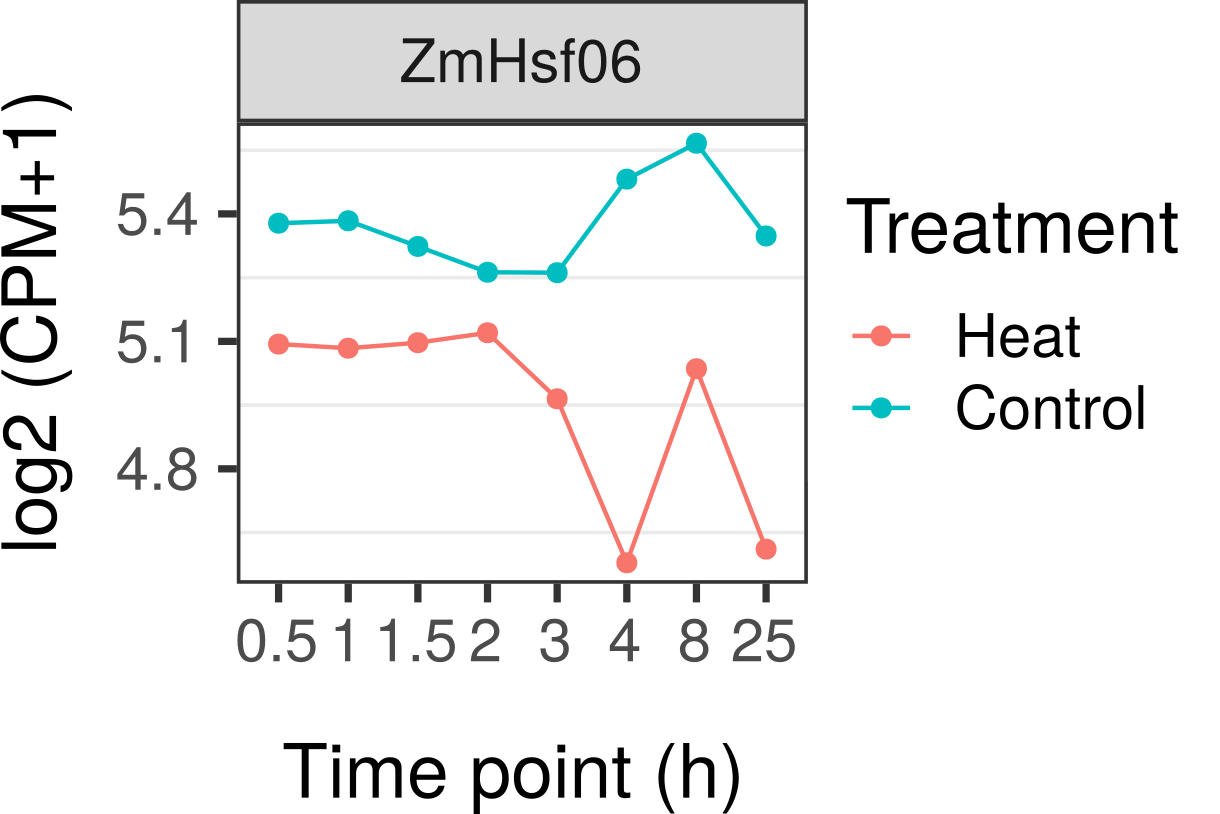
**

**Fig S4. Time-series expression of ZmHsf06 under control and heat conditions in six different time points.** Raw RNA-seq read counts were retrieved from a previous published paper [^14^](https://paperpile.com/c/G88hZq/Bcz1). Expression value was normalized to counts per million mapped reads (CPM).


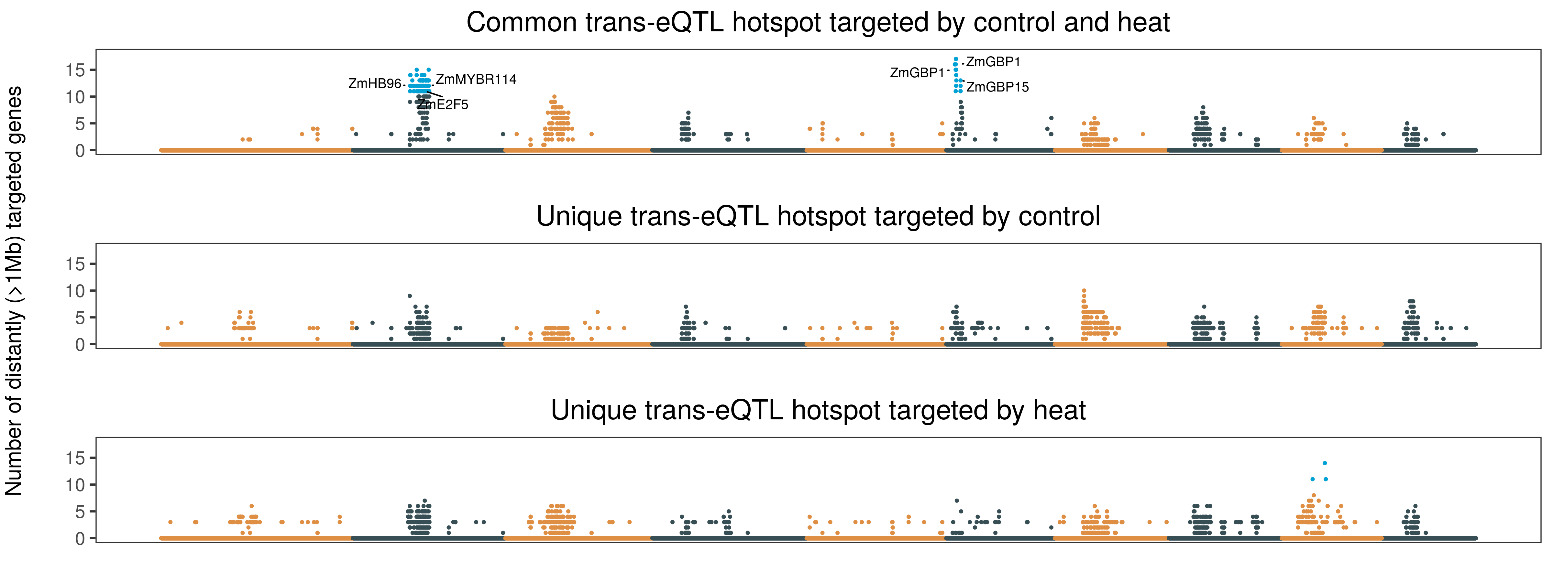


**Fig S5. *Trans*-eQTL hotspots identified in this study.** The genome was segmented into a 10kb bin. Each dot represents one 10kb and any SNP within each bin containing >= 3 targeted genes was retained. Any bin with more than 10 distantly targeted genes was considered as a *trans*-eQTL hotspot (labeled as blue). Known transcription factors were labeled for *trans*-eQTL hotspots.


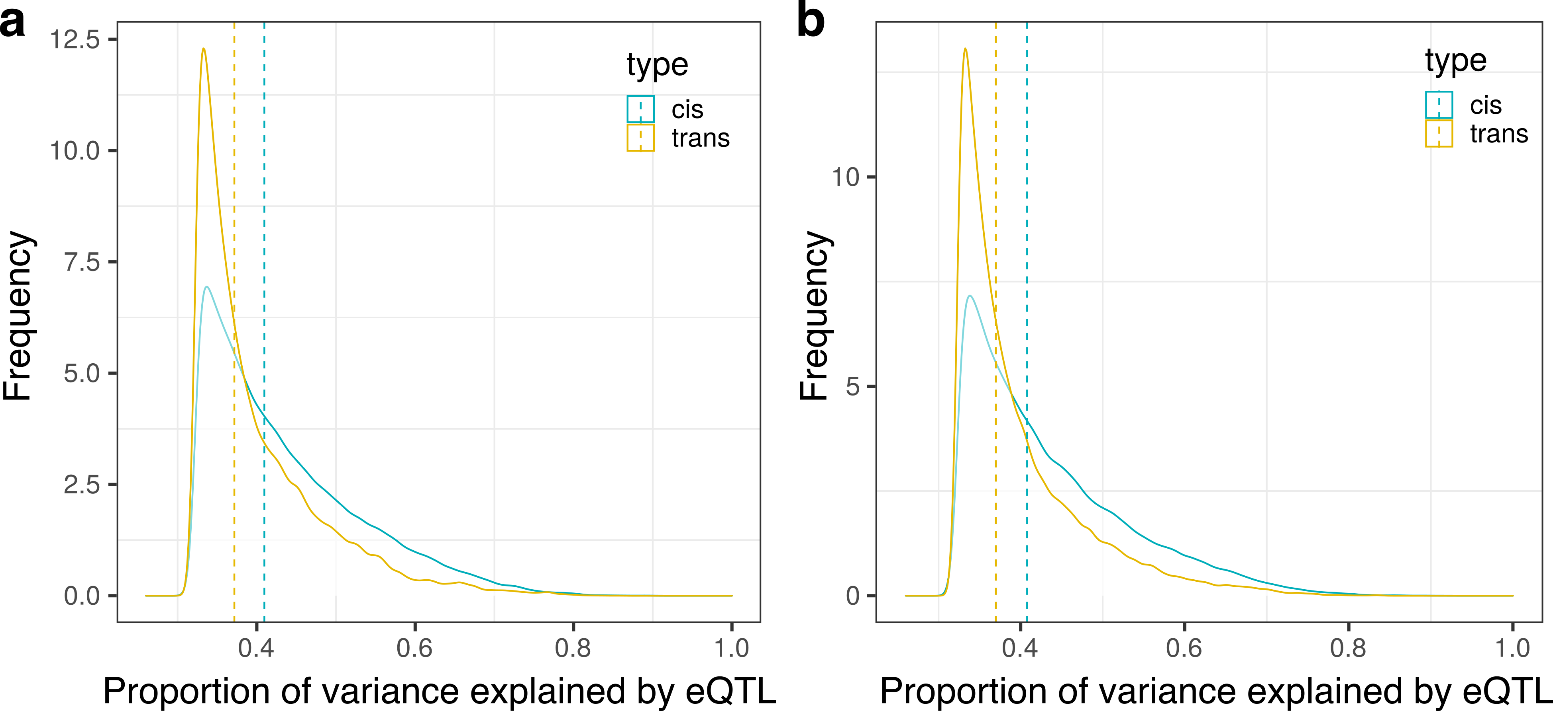


**Fig S6. Distributions of proportions of variance explained by *cis*-eQTLs and *trans*-eQTLs identified using (a) control and (b) heat expression data.** Dashed vertical lines indicate median value of either *cis*- or *trans*-eQTLs.


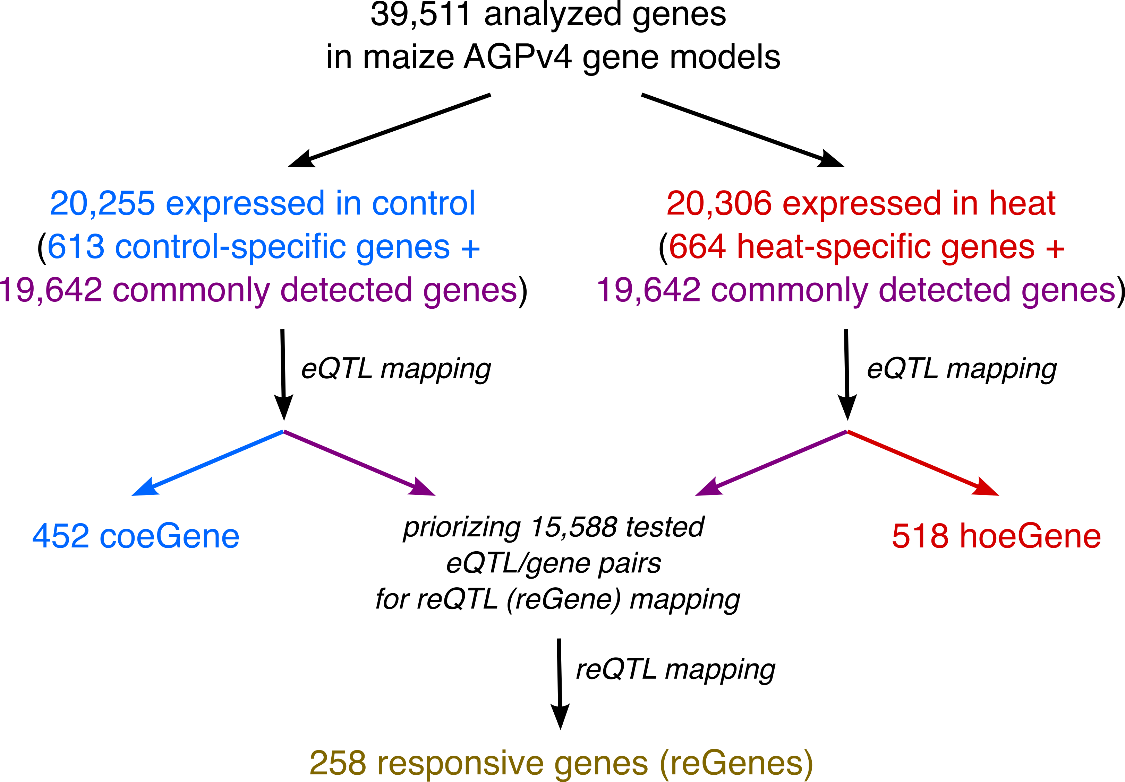


**Fig S7. Workflow for identifying reGenes and eGenes in control and heat.**

**
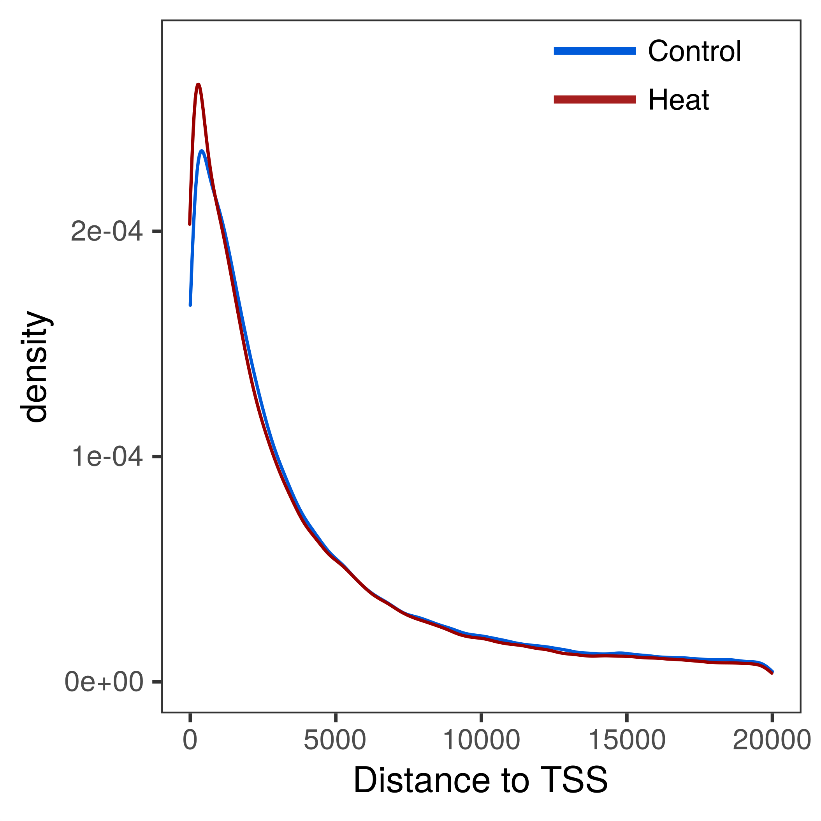
**

**Fig S8. Distance of MOA-seq footprints to TSS of genes.** The distance between MOA-seq TF footprint to TSS was limited to 20kb.

**
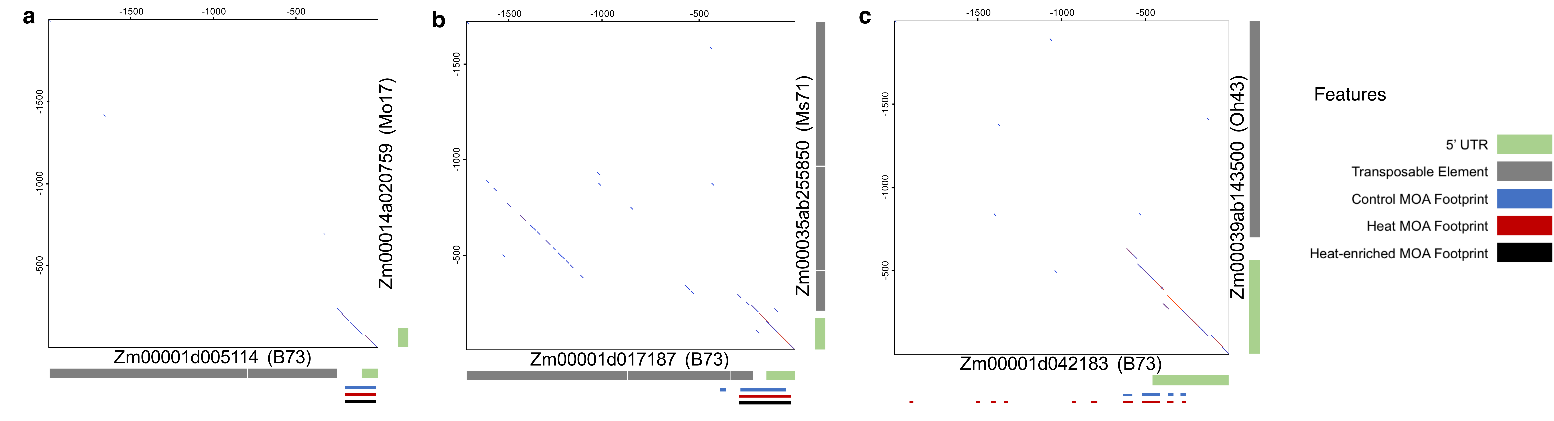
**

**Fig S9. Visualization of pairwise alignments between putative promoters in pairs of reGenes.** For each pair of promoter sequences that were assessed using the dual luciferase assay in protoplast systems we aligned the sequences of the two promoters. (a) *Zm00001d005114* and *Zm00014a020759;* (b) *Zm00001d017187* and *Zm00035ab255850;* (c) *Zm00001d042183 and Zm00039ab143500.* The dot plot shows the regions that are alignable as well as small insertions and deletions within these regions. The annotations of transposable elements from B73v5 and the other NAM genomes were used to document transposable elements.
