# Supplementary material for: Mapping responsive genomic elements to heat stress in a maize diversity panel
Source: Genome Biol. 2022 Nov 7;23:234. doi: 10.1186/s13059-022-02807-7 (PMC9639295; doi:10.1186/s13059-022-02807-7)
Supplement: Supplementary file 3 — Additional file 3. Review history. [file 13059_2022_2807_MOESM3_ESM.docx]

Review History

**First round of review**

**Reviewer 1**

**Were you able to assess all statistics in the manuscript, including the appropriateness of statistical tests used?**

Yes, and I have assessed the statistics in my report.

**Comments to author:**

In this article entitled "Mapping Responsive Genomic Elements to Heat Stress in a Maize Diversity Panel", the authors sought for expression QTL in response to heat stress in ~100 inbreed maize accessions and investigated their underlying genetic association for expression variation in response to heat stress and their mode of action (cis vs trans-acting eQTL). Subsequently, the authors detected that the differentially expressed genes are enriched with heat associated TF footprints. The manuscript is well written and the study is of interest to broader scientific community in this field. I outlined some of my major and minor concerns below.

Major comments:

1. The authors have mentioned a hierarchical clustering method to select a subset of 120 genotypes from 509 inbred lines from the WiDiv (Wisconsin Diversity) Panel. Further they retained a set f 102 genotypes which demonstrated congruence of SNP variants called from published genomic re-sequencing data and the RNA-seq data generated in this study. Hansey et at (2011) demonstrated substantial population structure for WiDiv panel. It is surprising that the author has not really talked about the genetic diversity within this population given the fact that earlier study mentioned eight substructure groups. The only place author mentioned about these substructures is in Figure 1a and corresponding result section mentioning the proportion of genetic variation explained by principle component of genotype matrix. More information is required about the genetic diversity among different substructure groups and the distribution of sample size across substructure groups which could down the road influence the eQTL analysis. The authors mentioned to use first five PCs along with some other factors as covariates. Does these PCs represent the structure and can control expression variance due to structured population?

2. The authors classified a QTL cis or trans based on the distance from the associated SNP with the focal gene. In line 609-610, the author mentioned that they defined any eQTL < 1 Mb distance within the focal gene was considered as cis category. There was no justification given in the manuscript why they chose 1Mb distance to classify between these two categories. The association of a SNP with regulatory elements of the focal gene would vary depending on the size of LD block for the given population. The average length of LD blocks might vary between different species and even in different natural populations of same species. Therefore, to define an eQTL as cis the author must provide substantial evidence or appropriate references to justify the definition of cis and trans eQTL.

Minor comments:

1. At Line 154 the authors mentioned "A comparison of the absolute difference of the first principal component values between control and heat condition based on transcriptome data and the correlation coefficient of the BLUP values for the photosynthetic traits revealed a significantly positive correlation, suggesting an overall 157 positive correlation between phenotypic and transcriptomic responses (Figure 2b; Figure S3). This statement is not very clearly written. It took a while to understand that the author has classified genotypes based on their transcriptome plasticity (large, medium and small) and asked for the correlation of BLUP values for the photosynthetic traits.

**Reviewer 2**

**Were you able to assess all statistics in the manuscript, including the appropriateness of statistical tests used?**

Yes, and I have assessed the statistics in my report.

**Comments to author:**

Liang and colleagues present a fascinating study of the genomic basis of genetic variation in heat stress response in maize. The authors first identify a genetically diverse panel of maize accessions, and document phenotypic diversity in the response of the photosystem to a moderate heat stress. Heritabilities for these traits are remarkably high, and the authors are able to map a small number of loci associated with these phenotypes via GWAS. The crux of the paper, however, lies in a transcriptomic assessment of heat response, integrating RNASeq, chromatin occupancy, and eQTL analysis. All of these analyses are performed to a high standard, and the results are interpreted in a conservative and appropriate way.

The major contribution of this work is the identification of genetic variants apparently driving GxE in gene expression response to heat stress. This is not a trivial undertaking, considering the nucleotide resolution achieved by intersecting eQTL and MOA-Seq data. Several promising candidates are validated directly via reporter assays.

I have no substantive criticism of this study, nor of the manuscript. It is exceptionally well done and should make a strong contribution to the literature that inspires future work in plants and non-plant systems.

Specific comments follow

Why is the concept of GxE never directly addressed? At base, this is a study of the molecular basis of regulatory GxE - I think the impact of the study could be greater by discussing some of the results in this context. Considering the substantial role that GxE plays in maize breeding (and human genetics, for that matter) I should think that researchers outside of the functional genomics field would appreciate this contribution.

Lines 73-74. I don't agree with the statement "it is not understood how novel TFBSs are created, or lost, at specific genes." A recent review by Hill, Zande, and Wittkopp (Nature Reviews Genetics 2020) offers some examples, as well as a broader framework for studying and interpreting natural genetic diversity in TFBS. Perhaps I am not fully appreciating what the authors mean by "understood" and more precise language might help.

Lines 159-162. I'm wary of cherry-picking GO terms from an enrichment analysis. Yes, three heat related terms are significant in that analysis. I would not characterize this as "many" and I would also like to see an statement acknowledging that many additional terms (some with much smaller p-values) with no apparent connection to heat stress were also identified.

Line 223-224. "reQTLs per se were more likely to be distributed…." This implies a statistical test, which is not reported here (not in the figure). If this claim cannot be backed by a formal test, I suggest re-wording (perhaps "appear to be" or "tend to be").

Line 575 - need a citation for GATK.

**Authors Response**

**Point-by-point responses to the reviewers’ comments:**

Reviewer #1: In this article entitled "Mapping Responsive Genomic Elements to Heat Stress in a Maize Diversity Panel", the authors sought for expression QTL in response to heat stress in ~100 inbreed maize accessions and investigated their underlying genetic association for expression variation in response to heat stress and their mode of action (cis vs trans-acting eQTL). Subsequently, the authors detected that the differentially expressed genes are enriched with heat associated TF footprints. The manuscript is well written and the study is of interest to broader scientific community in this field. I outlined some of my major and minor concerns below.

*Response: Thank you for the positive comments and for the useful suggestions. We appreciate the reviewer noting several things that were not explained clearly or where sufficient detail was not included.*

Major comments:

1. The authors have mentioned a hierarchical clustering method to select a subset of 120 genotypes from 509 inbred lines from the WiDiv (Wisconsin Diversity) Panel. Further they retained a set f 102 genotypes which demonstrated congruence of SNP variants called from published genomic re-sequencing data and the RNA-seq data generated in this study. Hansey et at (2011) demonstrated substantial population structure for WiDiv panel. It is surprising that the author has not really talked about the genetic diversity within this population given the fact that earlier study mentioned eight substructure groups. The only place author mentioned about these substructures is in Figure 1a and corresponding result section mentioning the proportion of genetic variation explained by principle component of genotype matrix. More information is required about the genetic diversity among different substructure groups and the distribution of sample size across substructure groups which could down the road influence the eQTL analysis. The authors mentioned to use first five PCs along with some other factors as covariates. Does these PCs represent the structure and can control expression variance due to structured population?

*Response: We appreciate this comment from the reviewer. We agree that the potential structure in this population is an important consideration and we have added more information in several ways. The short summary is that we do not think that population structure is a major contributing factor to our observed expression variation, especially after using the first five PCs as co-variates for the eQTL analysis. Below we provide additional detail and describe what additions/changes have been made.*

*First, we have provided more detail of the substructure classifications of the genotypes used in this study. We have updated the Supplemental Table S1 with substructure group information for each genotype employed in our study and we have specified the number of genotypes in each of the sub-populations to the legend for figure 1A. Second, we have added some context to the description of selection of genotypes to the methods section. By selecting a subset of genotypes that retain maximal genetic diversity from WiDiv we have reduced the overall population structure. While the full WiDiv population contains multiple highly related genotypes (which can result in higher levels of population structure), the selected subset of genotypes do not contain highly similar genotypes and will have reduced structure. Third, we have added some additional description of Figure 1A (which shows the first two PCs). The reviewer is correct that in this figure 1a we only used PC1 (the largest contributor) and PC2 (the second largest contributor) to* *illustrate the population structure in this panel. These two PCs do provide some separation of population subgroups but do not clearly distinguish them. The first five PCs can generally explain ~17% population structure (genetic variance) in this panel and these five PCs were incorporated in the eQTL model to control associations between expression values and allelic dosages that were mainly led by population structure. We did an analysis to assess the proportion of population structure explained by each additional PC and find highly diminished value in adding additional PCs (see figure below). Past the first 5 PCs there is not a strong gain of adding additional PCs and adding too many PCs will likely overcontrol population structure which can reduce the power of model, so we retained the first 5 PCs as covariates. Finally, we added some text when describing figure 2A. This figure shows a PCA of the RNA-seq data and within this analysis we do not find that population subgroups are a major factor in the clustering based on the first two PCs.*

2. The authors classified a QTL cis or trans based on the distance from the associated SNP with the focal gene. In line 609-610, the author mentioned that they defined any eQTL < 1 Mb distance within the focal gene was considered as cis category. There was no justification given in the manuscript why they chose 1Mb distance to classify between these two categories. The association of a SNP with regulatory elements of the focal gene would vary depending on the size of LD block for the given population. The average length of LD blocks might vary between different species and even in different natural populations of same species. Therefore, to define an eQTL as cis the author must provide substantial evidence or appropriate references to justify the definition of cis and trans eQTL.

*Response: This is a very good question and this is something we need to clarify in the manuscript. We understand the reviewer’s concern about LD block and the selection of threshold of distance for determining cis and trans eQTLs. If the distance threshold is close to LD decay in the population, we may gain an inaccurate estimate of trans-effect. There are two different answers to the question about why we are using* *1Mb. First, using data from the specific population used in this study, we observed that LD has significantly declined after ~100kb (r2 < 0.1) (Figure below). Our 1Mb threshold can generally eliminate the impact of LD on estimating trans-effects. Second, in a previous published paper (Azodi et al. 2020), the authors used genotypes from the same population (Wisconsin Diversity Panel) as our study to perform the eQTL mapping analysis and they had used 1Mb as the threshold for determining cis-eQTLs. A separate eQTL mapping study in using another maize population also considered 1Mb as the threshold for determining cis-eQTLs (Kremling KA et al. 2018). We have added this information to the manuscript to provide a better justification for the 1Mb distance.*

*Reference:*

*Azodi, C.B., Pardo, J., VanBuren, R., de Los Campos, G. and Shiu, S.H., 2020. Transcriptome-based prediction of complex traits in maize. The Plant Cell, 32(1), pp.139-151.*

*Kremling, K.A., Chen, S.Y., Su, M.H., Lepak, N.K., Romay, M.C., Swarts, K.L., Lu, F., Lorant, A., Bradbury, P.J. and Buckler, E.S., 2018. Dysregulation of expression correlates with rare-allele burden and fitness loss in maize. Nature, 555(7697), pp.520-523.*

Minor comments:

1. At Line 154 the authors mentioned "A comparison of the absolute difference of the first principal component values between control and heat condition based on transcriptome data and the correlation coefficient of the BLUP values for the photosynthetic traits revealed a significantly positive correlation, suggesting an overall 157 positive correlation between phenotypic and transcriptomic responses (Figure 2b; Figure S3). This statement is not very clearly written. It took a while to understand that the author has classified genotypes based on their transcriptome plasticity (large, medium and small) and asked for the correlation of BLUP values for the photosynthetic traits.

*Response: Thank the reviewer for mentioning this ambiguousness. We appreciate that the reviewer took the time to understand this result and we agree that this could be better explained. We have rephrased this expression as follows, “The absolute difference of PC1 between control and heat conditions reflected the* *transcriptome plasticity of each genotype and could be used to classify genotypes as having large, medium or small transcriptome responses. The BLUP for each genotype was determined as the genotypic effect after controlling experimental factors for the measured phenotype from either of conditions. We calculated correlations between BLUPs in both control and heat conditions for genotypes with large, medium or small transcriptome responses to heat stress (Figure 2b). Overall, for each measured phenotype, we observed an increased correlation coefficient with a larger transcriptome plasticity, suggesting a positive correlation between phenotypic and transcriptomic response in this study (Figure 2b; Figure S3).” Hopefully this more detailed description will help the reader understand this analysis better.*

Reviewer #2: Liang and colleagues present a fascinating study of the genomic basis of genetic variation in heat stress response in maize. The authors first identify a genetically diverse panel of maize accessions, and document phenotypic diversity in the response of the photosystem to a moderate heat stress. Heritabilities for these traits are remarkably high, and the authors are able to map a small number of loci associated with these phenotypes via GWAS. The crux of the paper, however, lies in a transcriptomic assessment of heat response, integrating RNASeq, chromatin occupancy, and eQTL analysis. All of these analyses are performed to a high standard, and the results are interpreted in a conservative and appropriate way.

The major contribution of this work is the identification of genetic variants apparently driving GxE in gene expression response to heat stress. This is not a trivial undertaking, considering the nucleotide resolution achieved by intersecting eQTL and MOA-Seq data. Several promising candidates are validated directly via reporter assays.

I have no substantive criticism of this study, nor of the manuscript. It is exceptionally well done and should make a strong contribution to the literature that inspires future work in plants and non-plant systems.

*Response: Thank you very much for the positive comments and the helpful suggestions.*

Specific comments follow

Why is the concept of GxE never directly addressed? At base, this is a study of the molecular basis of regulatory GxE - I think the impact of the study could be greater by discussing some of the results in this context. Considering the substantial role that GxE plays in maize breeding (and human genetics, for that matter) I should think that researchers outside of the functional genomics field would appreciate this contribution.

*Response: Thank you for this suggestion. We agree and have added this concept to both the introduction and to the discussion.*

Lines 73-74. I don't agree with the statement "it is not understood how novel TFBSs are created, or lost, at specific genes." A recent review by Hill, Zande, and Wittkopp (Nature Reviews Genetics 2020) offers some examples, as well as a broader framework for studying and interpreting natural genetic diversity in TFBS. Perhaps I am not fully appreciating what the authors mean by "understood" and more precise language might help.

*Response: Thank you for pointing this out. First, we need to be more clear in our phrasing and we have edited this statement in the manuscript. We were trying to point out that while we understand how SNPs could lead to differences in TFBSs we have less understanding how a novel cis-regulatory response (e.g. a* *heat response) arises for a gene. While SNPs could create a TFBS it might be difficult for this to occur at any gene as they would need to have a sequence very similar to a heat response element. Second, we have also included a citation to this really useful review.*

Lines 159-162. I'm wary of cherry-picking GO terms from an enrichment analysis. Yes, three heat related terms are significant in that analysis. I would not characterize this as "many" and I would also like to see an statement acknowledging that many additional terms (some with much smaller p-values) with no apparent connection to heat stress were also identified.

*Response: We agree that there can be lots to be wary about in terms of GO enrichment analyses. In this case we were not trying to do an exhaustive GO analysis to better understand heat responses but instead were simply trying to use this GO analyses to support the interpretation that we had successfully triggered a heat stress event. However, we understand the reviewers concern and have added some additional text to this section acknowledging that many other terms also exhibit enrichment.*

Line 223-224. "reQTLs per se were more likely to be distributed…." This implies a statistical test, which is not reported here (not in the figure). If this claim cannot be backed by a formal test, I suggest re-wording (perhaps "appear to be" or "tend to be").

*Response: Thank the reviewer for this point. We have rephrased the sentence as suggested. Now this sentence is "Compared to all cis-eQTLs identified in either of conditions or selected top significant cis-eQTLs for reQTLs identification, reQTLs per se tend to be to distributed around TSS (Transcriptional Start Site) regions of regulated genes (Figure 3b)."*

Line 575 - need a citation for GATK.

*Response: We have added a citation for GATK in the manuscript. The citation for GATK is "Van der Auwera, G. A. & O’Connor, B. D. Genomics in the Cloud: Using Docker, GATK, and WDL in Terra. (O’Reilly Media, 2020).".*

**Second round of review**

**Reviewer 1**

The authors addressed all my comments, and I am glad to see the appropriate references cited to address the issues. I do not have any follow-up comments. I believe that the study will significantly contribute to the field.

**Reviewer 2**

The authors have addressed all of my criticisms and suggestions.
